# Lipid compositional changes and oxidation status of ultra-high temperature treated Milk

**DOI:** 10.1186/s12944-018-0869-3

**Published:** 2018-10-02

**Authors:** Muhammad Ajmal, Muhammad Nadeem, Muhammad Imran, Muhammad Junaid

**Affiliations:** 1 Department of Dairy Technology, University of Veterinary and Animal Sciences, Lahore, Pakistan; 2 Institute of Home and Food Sciences, Faculty of Life Sciences, Government College University, Faisalabad, Pakistan

**Keywords:** UHT Milk, Fatty acid profile, Triglyceride profile, Induction period, Lipid oxidation

## Abstract

**Background:**

Milk fat is one of the complex fat and most sensitive biochemical compounds towards auto-oxidation. To enhance the shelf life, milk is subjected to Ultra-high Temperature (UHT) treatment followed by aseptic packaging. During the storage, several chemical and biochemical changes take place in lipid fraction of UHT milk. In current investigation, the effect of UHT treatment and storage was determined by making a comparison in fatty acid profile, triglyceride composition, organic acids and lipid oxidation of the thermally treated and stored milk with raw milk, which was not reported in earlier investigations.

**Methods:**

Raw milk samples were collected from the bulk storage facility of a dairy industry. The same milk was routed to UHT treatment and aseptically packaged samples were collected. The fatty acid profile, triglyceride composition, organic acids and lipid oxidation was determined in raw and UHT treated milk at 0, 30, 60 and 90 days. Fatty acid and triglyceride profile was determined on GC-MS while organic acids were determined by HPLC. For the measurement of induction period, professional Rancimat was used. Lipid oxidation was characterized through free fatty acids, peroxide value, anisidine value and conjugated dienes.

**Results:**

Compositional attributes of milk remain unchanged during the entire length of storage. Concentrations of short-chain fatty acids in raw and UHT milk were 10.49% and 9.62%. UHT treatment resulted in 8.3% loss of short-chain fatty acids. Up to 30 days, storage did not have any significant effect on fatty acid profile of UHT milk. Concentration of medium-chain fatty acids in raw and UHT treated milk was 54.98% and 51.87%. After 30, 60 and 90 days of storage, concentration of medium chain fatty acids was found 51.23%, 47.23% and 42.82%, respectively. Concentration of C_18:1_ and C_18:2_ in raw and UHT milk was 26.86% and 25.43%, respectively. The loss of C_18:1_ and C_18:2_ in UHT treatment was 5.32%. After 30, 60 and 90 days of storage, the concentrations of C_18:1_ and C_18:2_ were 24.6%, 21.06% and 18.66%, respectively. Storage period of 30 days was found non-significant, while noticeable variations were found in triglyceride profile of 60 and 90 days old samples of UHT milk. UHT treatment and storage period significantly affected the concentration of organic acids in milk. After UHT treatment, concentration of lactic acid, acetic acid, citric acid, pyruvic acid, formic acid, succinic acid and oxalic acid increased by 3.45, 0.66, 3.57, 0.68, 2.24, 2.16 and 1.63 mg/100 g. Effect of storage period on the production of organic acids in UHT milk was non-significant up to 30 days. After 60 days of storage period, the increase in concentration of lactic acid, acetic acid, citric acid, pyruvic acid, formic acid, succinic acid and oxalic acid was 3.79, 0.75, 4.69, 0.78, 2.83, 3.03 and 2.38 mg/100 g. After 90 days of storage period, the increase in concentration of lactic acid, acetic acid, citric acid, pyruvic acid, formic acid, succinic acid and oxalic acid was 7.3, 2.18, 9.96, 3.58, 11.37, 5.22 and 5.96%. Free fatty acids content of raw, UHT treated and 90 days old milk were 0.08%, 0.11% and 0.19%. UHT treated version of milk showed similar peroxide value. While, the storage remarkably affected the peroxide value. After 30, 60 and 90 days, peroxide value was 0.42, 0.62 and 1.18 (MeqO_2_/kg). Induction period of raw, UHT and stored milk was strongly correlated with peroxide value and fatty acid profile. Mean value of lipase activity in raw milk was 0.73 ± 0.06 μmoles/ml. UHT treatment significantly decreased the lipase activity. The lipase activity of milk immediately after the UHT treatment was 0.18 ± 0.02 μmoles/ml. Lipase activity of UHT milk after 30, 60 and 90 days of room temperature storage was 0.44 ± 0.03, 0.95 ± 0.07 and 1.14 ± 0.09 μmoles/ml. Color, flavor and smell score decreased through the storage of UHT milk for 90 days.

**Conclusion:**

The results of this investigation revealed that fatty acid and triglyceride profile changed after 60 and 90 days of storage. Production of organic acids led to the drop of pH and sensory characteristics in UHT milk during the long-term storage. Induction period can be successfully used for the determination of anticipatory shelf life of UHT milk.

## Background

In Ultra-high temperature (UHT) treatment, milk is exposed to a high temperature for a very short period of time to manufacture a commercially sterile product which does not require refrigeration storage [1]. Exposure of milk to UHT treatment and subsequent ambient storage leads to several biochemical changes such as proteolysis and lipolysis [2]. Lipid oxidation is one of the major reason for the spoilage of UHT milk. It not only decreases the nutritional value but also has a strong negative impact on sensory characteristics of UHT milk [3]. Volatile compounds generated from the breakdown of primary oxidation products induce oxidized flavor in UHT milk [4]. During lipid oxidation, fatty acids are broken down to oxidation products [5]. During lipid oxidation several biochemical changes take place which not only decrease the nutritional value but also lead to the production of oxidation products. Scientific evidences have shown that oxidation products may promote cancers and cardiovascular diseases [6]. Rate of lipid oxidation mainly depends upon the fatty acid composition [7]. Milk fat is highly complex, more than 400 fatty acids have been detected in it [8]. Milk fat contains about 70% saturated, 25% monounsaturated and 5% polyunsaturated fatty acids [9]. Physico-chemical characteristics of milk fat is determined by the triglycerides [10]. Functional, processing and quality characteristics of milk and dairy products are also influenced by the triglyceride profile [11]. During the storage, several biochemical changes take place in the fat fractions of milk, therefore, it is extremely important to study the effect of UHT treatment and storage on triglyceride profile, which is not previously investigated. Indigenous and bacterial lipases may cause hydrolysis of the triglyceride molecule resulting in the formation of free fatty acids and glycerol. Free fatty acid can also lead to the development of objectionable flavor in UHT milk. Bacterial lipases may survive the orthodox UHT treatment which can cause hydrolytic rancidity of milk during the storage. Earlier investigations have shown that UHT milk underwent lipid oxidation and developed of oxidized flavor during long term storage [12]. Preventing lipid oxidation in UHT milk is a major challenge for the dairy industries. UHT treatment may lead to the degradation of lactose which can lead to the formation of organic acids such as formic acid, acetic acid, pyruvic acid. Organic acids are responsible for the increased acidity of UHT milk during the storage [13]. The suitability of induction period to anticipate the shelf life of UHT milk is not previously investigated. Rancimat has been used for the estimation of shelf life of milk fat [14–16]. Lipid compositional changes and oxidation status of UHT milk needs further investigation. This study was planned to compare the proteolytic and lipolytic changes taking place in protein and lipid fractions of UHT milk using conventional and advanced techniques.

## Methods

### Materials and experimental plan

Raw milk samples were collected from the bulk storage facility of a dairy industry. The same milk was routed to UHT treatment and aseptically packaged samples were collected. Reagents used for this research work were procured from Sigma Aldrich, USA. UHT treated milk samples were stored at ambient conditions for a period of 90 days. Raw and UHT treated milk samples were analyzed for chemical and sensory characteristics at 0, 30, 60 and 90 days of storage.

### Milk composition and fatty acid profile

Milk composition was determined on a lactoscan. For the determination of fatty acid profile, fat was extracted from milk by centrifugation at 10,000 rpm for 10 min. Supernatant was dried over anhydrous Sodium Sulfate, the dried fat (50 mg) was taken in a screw capped test tubes followed by the addition of 2 ml solution of 15%Hydrogen Chloride in Methanol (15%, Fluka). Tubes were tightly closed and put in the heating block for 60 min with occasional stirring in first 20 min of incubation. After 60 min, tubes were cooled down to room temperature, 2 ml each deionized water and HPLC grade n-hexane were added, contents of the tubes were vortex at 2000 rpm for 2 min followed by the rest of 15 min. Supernatant was dried over anhydrous Sodium Sulfate, transferred to GC-MS (7890-B, Agilent Technologies). Specifications of column are: SP-2560 fused silica capillary column (LxI.D. 75mx0.18 mm, d_f_ 0.14 μm). Injector temperature was set at 150 °C. Column temperature was adjusted as: 50 °C for 1 min, temperature was then raised to 200 °C at the rate of 7 °C/min, temperature was then raised to 230 °C at the rate of 3 °C/min with a holding time of 18 min and total rum time was 44 min. Temperature of FID was set at 250 °C, flow rate of H_2_, O_2_ and He was set 40 ml, 400 ml and 2 ml/ min, respectively. FAME-37 mix standard (Supelco) were used for the identification and quantification of individual fatty acids [17].

### Triglycerides

Measurement of triglycerides was performed on GC-MS (7890-B, Agilent Technologies) fitted with RTX 65-TG capillary column, Restek, Bellefonte, CA, USA (30 m x id 0.25). Milk fat was dried over anhydrous sodium sulfate. The dried fat (50 mg) was then dissolve in *n*-hexane (1 ml) and 1 μL was injected into GC-MS through Auto Liquid Sampler (ALS). Injector temperature was adjusted to 350 °C, ramping of oven temperature was as: 250 °C for 2 min, then increase at 5 °C/min to 360 °C for 4 min. Detector temperature was 350 °C, hydrogen flow rate was 1.5 mL/min with a split ratio of 1:80 [18].

### Lipase activity

Lipase activity in milk samples was determined by pH static titratable method and expressed in μmoles i.e. the number of free fatty acids released from the triglyceride by lipases in 1 ml sample [19].

### Organic acids

Determination of organic acids in milk samples was performed as per method described [19]. For the extraction of organic acids, 4 parts of UHT milk were treated with 1 part of 10 mM sulfuric acid followed by centrifugation at 5000 rpm for min. Supernatant was filtered on a Whatman No.1 filter paper. Supernatant was injected into HPLC (HPLC-20 AD Prominence, Shimadzu, Kyoto, Japan) fitted with ion exchange column (Aminex HPX-87 H, 300 × 7.8 mm, BIO-RAD, Hercules, CA, USA). Detector was set at 214 nm, mobile phase was comprised of aqueous 0.5% (*w*/*v*) (NH4)2HPO4 (0.038 M) – 0.2% (*v*/v) acetonitrile (0.049 M) adjusted to pH 2.24 with H3PO4 with a flow rate of 0.5 ml/min. Standards of organic acids were purchased from Sigma-Aldrich.

### Lipid oxidation

Free fatty acids, peroxide value and anisdine value were determined at 0, 30, 60 and 90 days of storage period following the standard methods. Milk sample 2 ml was taken in 50 mL test tube and mixed with 18 mL of 3.86% HCLO_4_. The sample was homogenized for 15 s and to prevent the oxidation of lipid, butylated hydroxytoluene (50 μL, in 4.5% ethanol) was added. After homogenization, contents were filtered through whatman filter paper no. 1. Filtrate (2 ml) was mixed with 2 ml of 20 mM TBA in distilled water and incubated for 15–17 h in dark at room temperature. Absorbance was determined at 532 nm. The TBARS was denoted as mg of malondialdehyde/g of milk [20].

### Induction period

For the determination of induction period, fat from milk was extracted by standard method [21]. Sample 2.5 g was taken in the reaction vessels, temperature and rate of oxygen was set at 120 °C and 20 l/hr.

### Sensory evaluation

Sensory evaluation of milk samples was performed in a sensory evaluation laboratory at 25 °C, a trained panel of judges was used for the evaluation of color, flavor and smell [22].

### Statistical analysis

This research work was planned in a completely randomized design (CRD). Sampling stages were considered as treatments and replicated at least five times. Results were reported as Mean ± SD while for the estimation of significant difference among the treatments, Duncan Multiple Range Test was used. Data were analyzed on SAS 9.1 software [23].

## Results and discussion

Table 1 describes the results of chemical composition of raw milk, UHT treated and stored samples for 90 days. The difference in fat, protein and SNF content of raw and UHT treated milk was due to the standardization of milk at3.5% fat content. Fat, protein and SNF content non-significantly decreased during the storage of 90 days. The pH of 90 days old samples of UHT milk was significantly higher than raw milk, UHT treated, 30 and 60 days old milk samples. Compositional attributes of UHT milk have been studied in earlier investigations. Hassan et al. [24] studied the physico-chemical characteristics of UHT milk for a period of 180 days and non-significant changes were observed in the compositional attributes with little increase in acidity. Martins and van Boekel [25] also reported similar results on the chemical composition of UHT milk. Anema and Li [26] reported that pH of UHT milk decreased during the long-term storage.

**Table 1:** Effect of UHT Treatment and Storage on Chemical Composition of Milk

Stage	Fat%	Protein%	SNF%	pH	Acidity%
Raw Milk	4.58 ± 0.08^a^	3.25 ± 0.13^a^	8.65 ± 0.10^a^	6.82 ± 0.17^a^	0.12 ± 0.02^c^
UHT Milk	3.52 ± 0.05^b^	3.22 ± 0.09^a^	8.62 ± 0.16^a^	6.79 ± 0.09^a^	0.12 ± 0.01^c^
30 Days Stored UHT Milk	3.51 ± 0.12^b^	3.22 ± 0.07^a^	8.61 ± 0.19^a^	6.78 ± 0.06^a^	0.12 ± 0.05^c^
60 Days Stored UHT Milk	3.45 ± 0.03^b^	.318 ± 0.04^a^	8.56 ± 0.22^a^	6.65 ± 0.18^a^	0.15 ± 0.04^b^
90 Days Stored UHT Milk	3.41 ± 0.15^b^	3.11 ± 0.02^b^	8.42 ± 0.02^b^	6.51 ± 0.24^c^	0.18 ± 0.01^a^

If the means in a column are expressed by a same letter these are non-significant (*p* > 0.05)

### Fatty acid profile

Fatty acid profile of raw, UHT treated and aseptically packaged milk samples stored for 90 days has been shown in Table 2. UHT treatment and storage period significantly affected the fatty acid profile of milk. Concentration of short-chain, medium-chain and long-chain fatty acids decreased during the UHT treatment. Concentrations of short-chain fatty acids in raw and UHT milk were 10.49% and 9.62% while UHT treatment lead to a loss of about 8.3% of short-chain fatty acids. The effect of UHT processing on fatty acid profile of milk is described in literature [27]. However, the effect of storage period on fatty acid profile of UHT milk during ambient storage is not reported. According to the results, concentrations of short-chain fatty acids in raw milk and UHT milk were 10.6% and 9.28%. Up to 30 days, storage did not have any significant effect on fatty acid profile of UHT milk. Major changes were recorded in fatty acid profile, when milk samples were analyzed at 60 and 90 days of storage. After 30, 60 and 90 days of storage, concentration of short-chain fatty acids was 9.39%, 8.53% and 7.75%. Short-chain fatty acid are highly significant from the flavor and sensory characteristics viewpoints, decline in their concentration may have a negative impact on flavor perspectives of milk and dairy products [14]. Vazquez-Landaverde et al. [28] reported that cheddar cheese having higher concentration of short-chain fatty acids had better sensory characteristics. Concentration of medium-chain fatty acids in raw and UHT treated milk was 54.98% and 51.87%. After 30, 60 and 90 days of storage, concentration of medium chain fatty acids 51.23%, 47.23% and 42.82%. Concentration of C_18:1_ and C_18:2_ in raw and UHT milk was 26.86% and 25.43%, respectively. The loss of C_18:1_ and C_18:2_ in UHT treatment was 5.32%. After 30, 60 and 90 days of storage, the concentrations of C_18:1_ and C_18:2_ were 24.6%, 21.06% and 18.66%, respectively. Pestana et al. [27] studied the effect of UHT pasteurization and UHT treatment of milk and UHT treatment significantly affected the fatty acid profile, while, pasteurization had a non-significant effect on fatty acid profile. Unsaturated fatty acids (C_18:1_ and C_18:2_) are prone to auto-oxidation. [29] compared the rate of oxidation in C_18:1_ and C_18:2_ and their study disclosed that rate of oxidation in C_18:2_ was ten times faster than C_18:1_. The effect of UHT treatment on fatty acid profile of milk is studied only in a limited way Topçu et al. [30]. Storage induced oxidative deterioration in heat treated milk during the storage. UHT milk is stored at ambient temperature that can accelerate the auto-oxidation in UHT milk [3]. Lipid oxidation is a serious problem of UHT milk that not decreases the nutritional value but also reduces the sensory prospects [31]. Choe [32] reported that oleic acid is the dominant unsaturated fatty acid in milk. Elevated storage temperature can accelerate the auto-oxidation in UHT milk.

**Table 2:** Impact of UHT Treatment and Storage on Fatty Acid Profile of Milk

Fatty Acid Profile mg/g	Raw Milk	Immediately After UHT Treatment	30-Days	60-Days	90-Days
C_4:0_	3.79 ± 0.06^a^	3.44 ± 0.05^b^	3.39 ± 0.03^b^	3.12 ± 0.08^c^	2.88 ± 0.13^d^
C_6:0_	2.52 ± 0.26^a^	2.38 ± 0.07^b^	2.35 ± 0.05^b^	2.19 ± 0.04^c^	2.04 ± 0.17^d^
C_8:0_	1.41 ± 0.15^a^	1.29 ± 0.03^b^	1.22 ± 0.11^b^	1.08 ± 0.03^c^	1.02 ± 0.08^c^
C_10:0_	2.77 ± 0.05^a^	2.51 ± 0.09^b^	2.43 ± 0.10^b^	2.14 ± 0.16^c^	1.95 ± 0.02^d^
C_12:0_	3.11 ± 0.09^a^	2.91 ± 0.14^b^	2.84 ± 0.17^b^	2.61 ± 0.07^c^	2.44 ± 0.01^c^
C_14:0_	10.22 ± 0.23^a^	9.44 ± 0.25^b^	9.18 ± 0.16^b^	8.22 ± 0.28^c^	7.56 ± 0.25^d^
C_16:0_	25.38 ± 0.77^a^	24.16 ± 0.42^b^	24.10 ± 0.35^b^	22.74 ± 0.17^c^	21.53 ± 0.64^d^
C_18:0_	16.27 ± 0.19^a^	15.36 ± 0.18^b^	15.11 ± 0.33^b^	13.66 ± 0.09^c^	11.29 ± 0.15^d^
C_18:1_	24.69 ± 0.55^a^	23.29 ± 0.33^b^	22.87 ± 0.66^b^	20.47 ± 0.33^c^	18.52 ± 0.13^d^
C_18:2_	2.17 ± 0.05^a^	1.98 ± 0.12^b^	1.73 ± 0.02^c^	0.59 ± 0.14^d^	0.14 ± 0.01^e^

Means having same letter in a row are non-significant (*p* > 0.05)

### Triglyceride profile of UHT Milk

Fat content of bovine milk ranges from 2.5–6.5% that is comprised of triglycerides [33]. Fat content and fatty acid composition considerably vary from breed to breed, season and stage of maturity [34]. Milk fat is regarded as one of the most complex fat. More than 100 kinds of triglyceride species have been found in milk fat [35]. In some studies, fatty acid profile of UHT is investigated, however, no investigation is reported regarding the effect of UHT treatment and storage on triglyceride profile of UHT milk [36]. Physico-chemical and functional properties of milk fat are largely determined by the triglyceride composition that is highly significant for the quality and shelf stability of dairy products and absorption efficiency of fatty acids in the body may is also affected by the triglyceride profile [37]. The results of triglyceride profile of raw, fresh and stored UHT milk are given in Table 3. UHT treatment induced significant changes in triglyceride profile of milk. Storage duration up to 30 days was non-significant, while noticeable variations were found in triglyceride profile of 60 and 90 days old samples of UHT milk. Heat, moisture, metal ions and lipases have been recognized as catalysts for the hydrolysis of triglycerides. Milk contains about 87% water, lipases are also present in milk. Bacterial lipases usually survive the orthodox UHT treatment, cleaves the bond between the fatty acids and glycerol leading to the formation free fatty acids, that consequences in the reduction of triglyceride content of milk. Determination frequencies showing higher content of free fatty acids showed lower number of triglycerides. Triglycerides are the key component of fat; all triglycerides have not same correlation with the fat content of milk. TAG _36:1_ and TAG _42:1_ have strong correlation with total fat content of milk, these two TAGS accounts for small contribution in milk fat [38]. Milk fat is comprised of 97–98% triglycerides [39]. UHT milk is usually toned and standardized at 3.5% fat content, therefore, TAG profile of UHT milk may be different from raw milk. Beccaria et al. [40] studied the correlation between TAG and fatty acid profile in milk and stereospecific analyses were performed to determine the magnitude of fatty acids at *sn*-1, *sn*-2 and *sn*-3 positions of TAG. Strong connections were recorded in the content of fatty acid at all the three positions and content of similar fatty acids on the intact TAG.

**Table 3:** Effect of UHT Treatment and Storage on Triglyceride Profile of Milk

Triglyceride Profile mg/g	Raw Milk	Immediately After UHT Treatment	30-Days	60-Days	90-Days
TAG _C24:0_	0.20 ± 0.01^a^	0.14 ± 0.01^b^	0.12 ± 0.02^b^	0.08 ± 0.02^c^	0.03 ± 0.01^d^
TAG _C26:1_	0.89 ± 0.05^a^	0.81 ± 0.03^b^	0.79 ± 0.13^b^	0.64 ± 0.08^c^	0.52 ± 0.05^d^
TAG _28:0_	1.29 ± 0.08^a^	1.15 ± 0.07^b^	1.12 ± 0.19^b^	0.98 ± 0.04^c^	0.85 ± 0.11^d^
TAG _C28:1_	0.14 ± 0.02^a^	0.12 ± 0.03^b^	0.11 ± 0.01^b^	0.06 ± 0.02^c^	0.03 ± 0.01^d^
TAG _30:0_	1.91 ± 0.06^a^	1.79 ± 0.11^b^	1.75 ± 0.05^b^	1.62 ± 0.14^c^	1.54 ± 0.02^d^
TAG _C30:1_	0.28 ± 0.02^a^	0.24 ± 0.05^b^	0.22 ± 0.02^b^	0.17 ± 0.04^c^	0.13 ± 0.01^d^
TAG _32:0_	3.35 ± 0.12^a^	3.11 ± 0.09^b^	3.07 ± 0.08^b^	2.92 ± 0.16^c^	2.61 ± 0.19^d^
TAG _C32:1_	1.74 ± 0.06^a^	1.55 ± 0.06^b^	1.52 ± 0.03^b^	1.31 ± 0.09^c^	1.22 ± 0.13^d^
TAG _34:0_	7.56 ± 0.10^a^	7.12 ± 0.14^b^	7.08 ± 0.28^b^	6.91 ± 0.27^c^	6.58 ± 0.04^d^
TAG _C34:1_	3.41 ± 0.09^a^	3.18 ± 0.05^b^	3.14 ± 0.02^b^	2.89 ± 0.012^c^	2.41 ± 0.02^d^
Tab. _36:0_	11.39 ± 0.35^a^	10.82 ± 0.16^b^	10.77 ± 0.31^b^	10.17 ± 0.49^c^	9.42 ± 0.37^d^
TAG _C36:1_	3.67 ± 0.03^a^	3.49 ± 0.07^b^	3.11 ± 0.02^b^	2.88 ± 0.06^c^	2.27 ± 0.01^d^
TAG _38:0_	14.66 ± 0.45^a^	14.18 ± 0.33^b^	14.11 ± 0.56^b^	13.42 ± 0.71^c^	12.54 ± 0.43^d^
TAG _C38:1_	4.77 ± 0.13^a^	4.22 ± 0.16^b^	3.66 ± 0.08^b^	2.99 ± 0.03^c^	2.38 ± 0.15^d^
TAG _C40:2_	10.55 ± 0.18^a^	9.76 ± 0.64^b^	9.71 ± 0.73^b^	8.66 ± 0.20^c^	7.44 ± 0.26^d^
TAG _C42:1_	5.11 ± 0.38^a^	4.62 ± 0.28^b^	4.55 ± 0.21^b^	4.19 ± 0.07^c^	3.78 ± 0.03^d^
TAG _C44:1_	4.62 ± 0.19^a^	4.21 ± 0.04^b^	4.17 ± 0.08^b^	3.48 ± 0.06^c^	3.17 ± 0.02^d^
TAG _C46:1_	5.16 ± 0.17^a^	4.58 ± 0.02^b^	4.51 ± 0.17^b^	4.13 ± 0.22^c^	3.64 ± 0.04^d^
TAG _C48:3_	7.66 ± 0.21^a^	6.74 ± 0.12^b^	7.63 ± 0.09^b^	7.23 ± 0.09^c^	6.74 ± 0.05^d^
TAG _C50:5_	11.27 ± 0.61^a^	11.25 ± 0.16^b^	11.24 ± 0.33^b^	10.51 ± 0.31^c^	9.42 ± 0.27^d^
TAG _C52:1_	10.24 ± 0.34^a^	10.22 ± 0.27^b^	10.21 ± 0.67^b^	9.77 ± 0.06^c^	8.27 ± 0.15^d^
TAG _C54:6_	2.66 ± 0.03^a^	2.65 ± 0.04^b^	2.61 ± 0.13^b^	2.27 ± 0.01^c^	2.11 ± 0.02^d^

In a row, if means are expressed by a different letter, that are statistically significant (*p* < 0.05)

### Organic acids

During UHT treatment, lactose endures several chemical changes such as Maillard Reaction and formation of formic acid, pyruvic acid, acetic acid etc. Organic acids produced during UHT treatment are largely responsible for the increased acidity of UHT milk [41]. Quality of raw milk, thermal processing and storage conditions may affect the concentration of organic acids in UHT treated milk [42]. Results of organic acids in raw, UHT milk at different stage of storage have been shown in Table 4. In current investigation, lactic acid, acetic acid, citric acid, pyruvic acid, formic acid, succinic acid and oxalic acid were determined in raw milk, UHT milk, 30, 60 and 90 days old UHT milk. UHT treatment and storage period significantly affected the concentration of organic acids in milk. After UHT treatment, concentration of lactic acid, acetic acid, citric acid, pyruvic acid, formic acid, succinic acid and oxalic acid increased by 3.45, 0.66, 3.57, 0.68, 2.24, 2.16 and 1.63 mg/100 g. Storage duration up to 30 days was non-Significant, after 60 days of storage period, the increase in concentration of lactic acid, acetic acid, citric acid, pyruvic acid, formic acid, succinic acid and oxalic acid was 3.79, 0.75, 4.69, 0.78, 2.83, 3.03 and 2.38 mg/100 g. After 90 days of storage period, the increase in concentrations of lactic acid, acetic acid, citric acid, pyruvic acid, formic acid, succinic acid and oxalic acid was 7.3, 2.18, 9.96, 3.58, 11.37, 5.22 and 5.96%, respectively. Claeys et al. [43] reported that concentrations of citric acid in pasteurized milk was 1439 mg/L. Heat treatment may have an impact on citric acid content in milk, however, detailed investigation on this aspect should be performed [42]. Islam et al. [44] studied the concentration of organic acids in raw, pasteurized and UHT milk and they reported that concentrations of organic acids increased in the storage [44]. Formic acid in heated milk is recognized as advanced product of Maillard reaction and amount of formic acid in sterilized milk was 150 times greater than normally pasteurized milk [45]. The decline in pH of UHT milk is mainly due to the production of formic acid and UHT milk having higher level of formic acid had lower pH and more acidity [43]. Organic acids in UHT milk are mainly produced from the degradation of lactose and amino acids with heat treatment as the major factor determining their production. Presently, there is no literature available on the magnitude of organic acids produced during the long-term storage of UHT milk. In current investigation, the production of organic acids in UHT milk was assessed for a period of 3 months. Walstra et al. [46] described that UHT treatment may lead to the degradation of lactose to organic acids.

**Table 4:** Effect of UHT Treatment and Storage on Organic Acids in Milk

Organic Acids mg/100 g	Raw Milk	Immediately After UHT Treatment	30-Days	60-Days	90-Days
Lactic Acid	60.43 ± 0.57^c^	63.88 ± 0.09^b^	64.17 ± 0.04^b^	64.22 ± 0.41^b^	67.73 ± 0.82^a^
Acetic Acid	2.96 ± 0.04^c^	3.62 ± 0.13^b^	3.66 ± 0.06^b^	3.71 ± 0.50^b^	5.14 ± 0.32^a^
Citric Acid	88.57 ± 0.74^c^	92.34 ± 0.69^b^	93.11 ± 0.43^b^	93.44 ± 0.98^b^	98.53 ± 0.26^a^
Pyruvic Acid	3.61 ± 0.08^c^	4.29 ± 0.10^b^	4.32 ± 0.24^b^	4.39 ± 0.06^b^	7.19 ± 0.18^a^
Formic Acid	84.27 ± 0.43^c^	86.51 ± 0.16^b^	86.97 ± 0.33^b^	87.10 ± 0.51^b^	95.64 ± 0.13^a^
Succinic Acid	20.16 ± 0.15^c^	22.67 ± 0.35^b^	23.14 ± 0.28^b^	23.19 ± 0.40^b^	25.38 ± 0.61^a^
Oxalic Acid	4.31 ± 0.02^c^	5.94 ± 0.12^b^	6.19 ± 0.21^b^	6.24 ± 0.31^b^	10.27 ± 0.23^a^

In a row, if means are expressed by a different letter, that are statistically significant (*p* < 0.05)

### Lipid oxidation

**Fig. 1:**
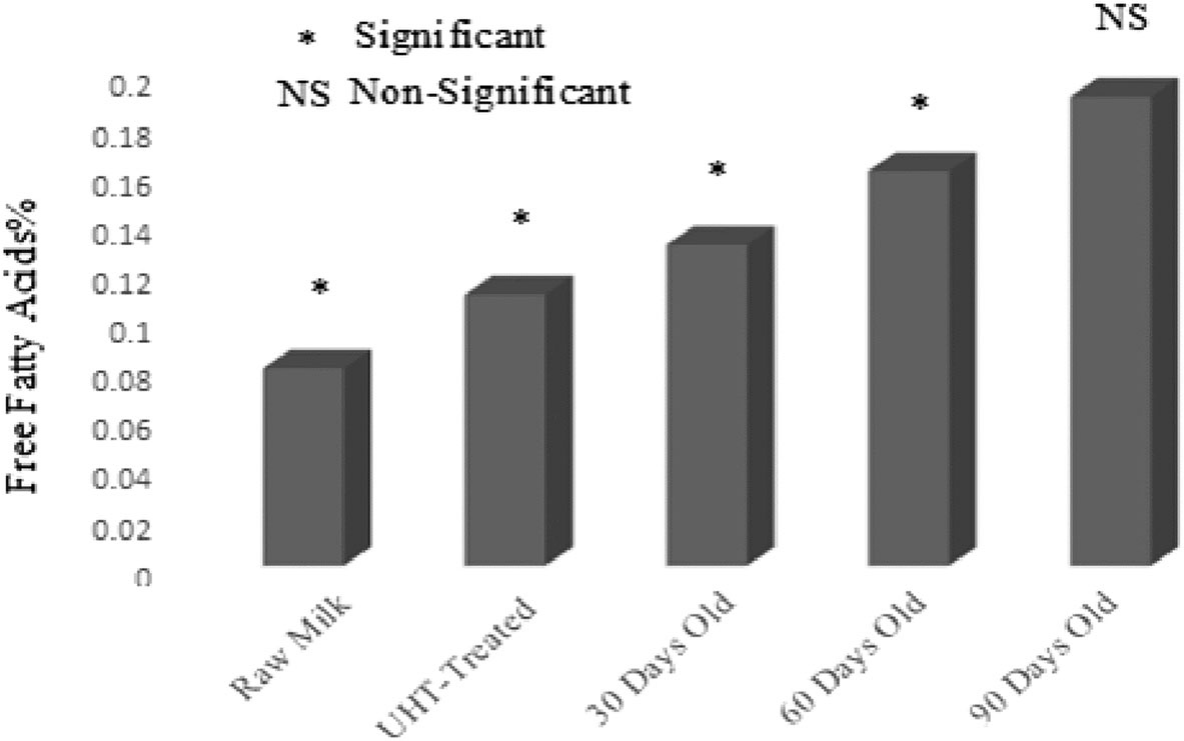
Effect of UHT Treatment and Storage on Free Fatty Acids

**Fig. 2:**
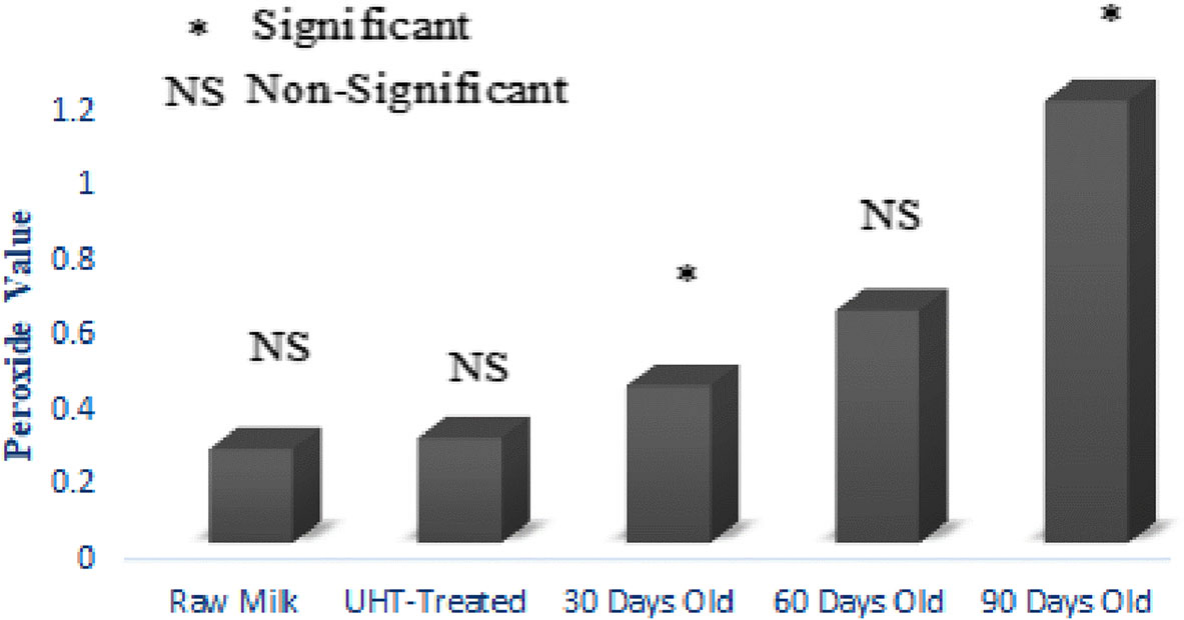
Effect of UHT Treatment and Storage on Peroxide Value

**Fig. 3:**
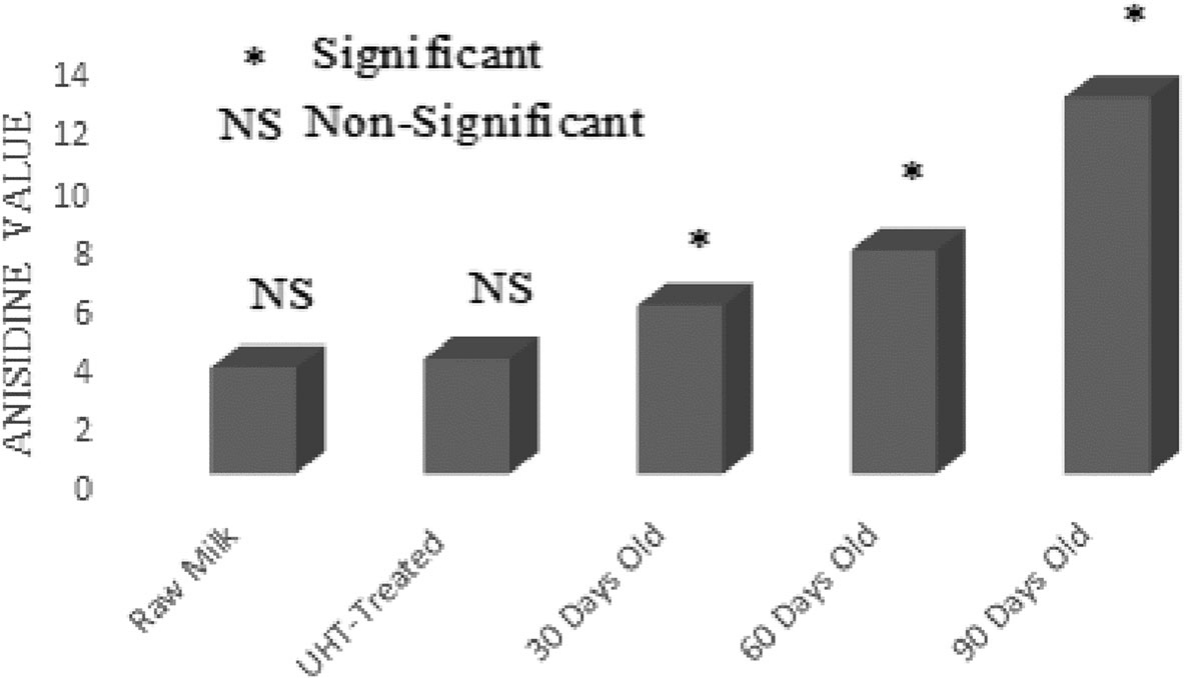
Effect of UHT Treatment and Storage on Anisidine Value

**Fig. 4:**
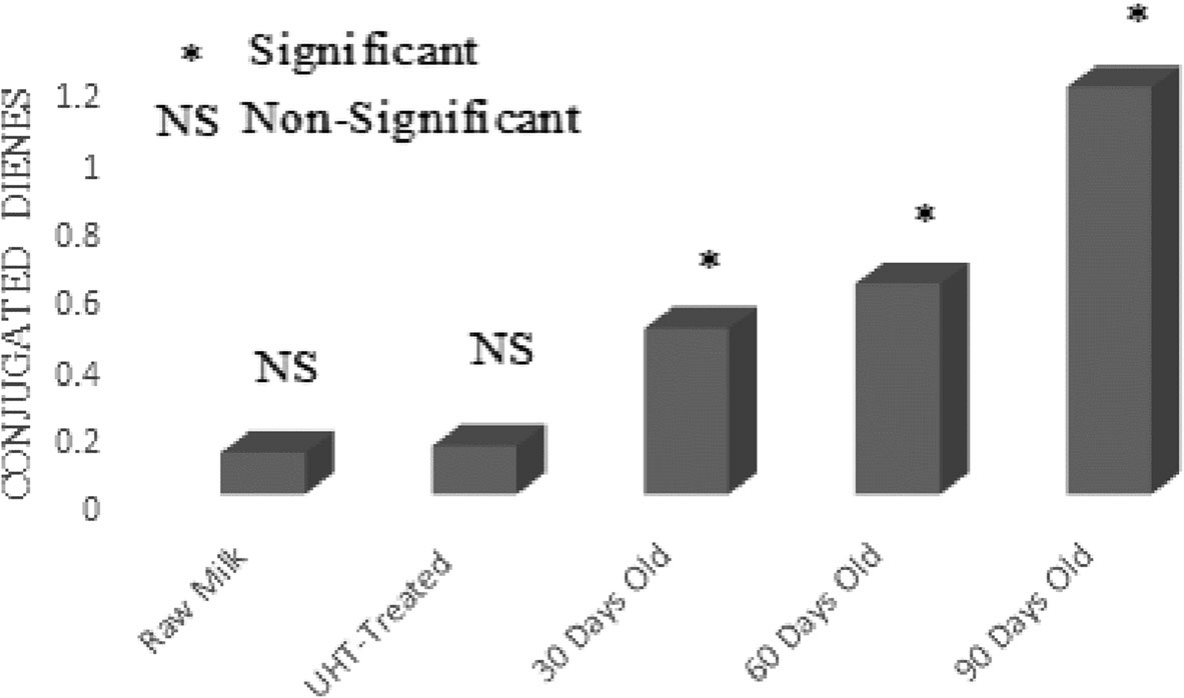
Effect of UHT Treatment and Storage on Conjugated Dienes

UHT treatment is designed to make extended life milk that can be stored at room temperature for a fairly large period of time. In current investigation, the effect of UHT treatment and storage period on lipid oxidation of UHT milk was studied in detail. Free fatty acids, peroxide value, anisidine value and conjugated dienes were used as parameters to quantify the magnitude of lipid oxidation in raw and UHT treated milk (Figs. 1, 2, 3 and 4). Lipid oxidation is one of the major cause of spoilage in UHT milk which leads to the development of oxidized flavor and oxidation products. It is scientifically established that ingestion of oxidation products can lead to cardiovascular diseases, cancer and atheorgenesis [47]. During the storage, UHT milk experienced oxidative and hydrolytic rancidity. For the measurement of hydrolytic rancidity, free fatty acids were used. Free fatty acids in milk fat lead to the development of off-flavor [48]. Lipases of bacterial and milk origin are mainly responsible for the hydrolysis of triglycerides and generation of free fatty acids. Poor quality raw milk has more number of lipases leading to the generation of more fatty acids. European Union has established a maximum limit of 0.2% free fatty acids in foods. In this research work, free fatty acids content of raw, UHT treated and 90 days old milk samples were 0.08%, 0.11% and 0.19%, respectively. In food matrix, free fatty acids perform two types of negative activities; lead to the development of objectionable flavors and also accelerate the oxidative deterioration of fats and oils [49]. Investigations have shown that milk fat is susceptible to auto-oxidation [14]. Milk fat contains about 23–25% oleic acid and 1.5–2% linoleic acids, the latter has 100 times more susceptible to auto-oxidation as compared to the former [29]. For the assessment of oxidative deterioration and oxidation status of foods, peroxide value is the method of choice for the researchers working in the field of analytical chemistry and food science [50]. UHT treatment had no effect on peroxide value of milk. While, the storage duration remarkably affected the peroxide value. After 30, 60 and 90 days of storage duration, peroxide value was 0.42, 0.62 and 1.18 (MeqO_2_/kg). The rise in peroxide value was due to the breakdown on fatty acids into oxidation products and analysis intervals showing more peroxide value also showed pronounced changes in the fatty acids profile. Chavan et al. [14] a non-significant effect of pasteurization on peroxide value of milk. For the estimation of primary oxidation products produced during the storage of UHT milk, conjugated dienes were determined. Values of conjugated dienes were not affected by the UHT treatment. However, the conjugated dienes considerably increased as the storage progressed. After 30, 60 and 90 days storage of UHT treated milk, conjugated dienes were 0.48, 0.61 and 1.18. For the determination of primary oxidation products in milk fat, Chavan et al. [15] used conjugated dienes. During auto-oxidation of fats and oils, ketones, alcohols and aldehydes are produced [51]. Anisidine value is used to measure the number of aldehydes especially 2-alkenals that are produced during the course of auto-oxidation. Anisidine value of raw and UHT milk were indifferent, however, storage conditions strongly influence intensified the anisidine value. Anisidine value of raw milk and 90 days old raw milk was 3.59 and 12.73, respectively. Topçu et al. [30] studied the effect of heat treatment on anisidine value of cow and buffalo milk, they reported that heat treatment did not have any significant influence on anisidine value while, it considerably increased during the storage of 90 days. TBARS values raw and UHT milk were 0.45 and 0.47 MDA/kg. After 30, 60 and 90 days of storage duration, TBARS value of UHT milk 0.51, 1.19 and 1.81 MDA/kg.

### Induction period

**Fig. 5:**
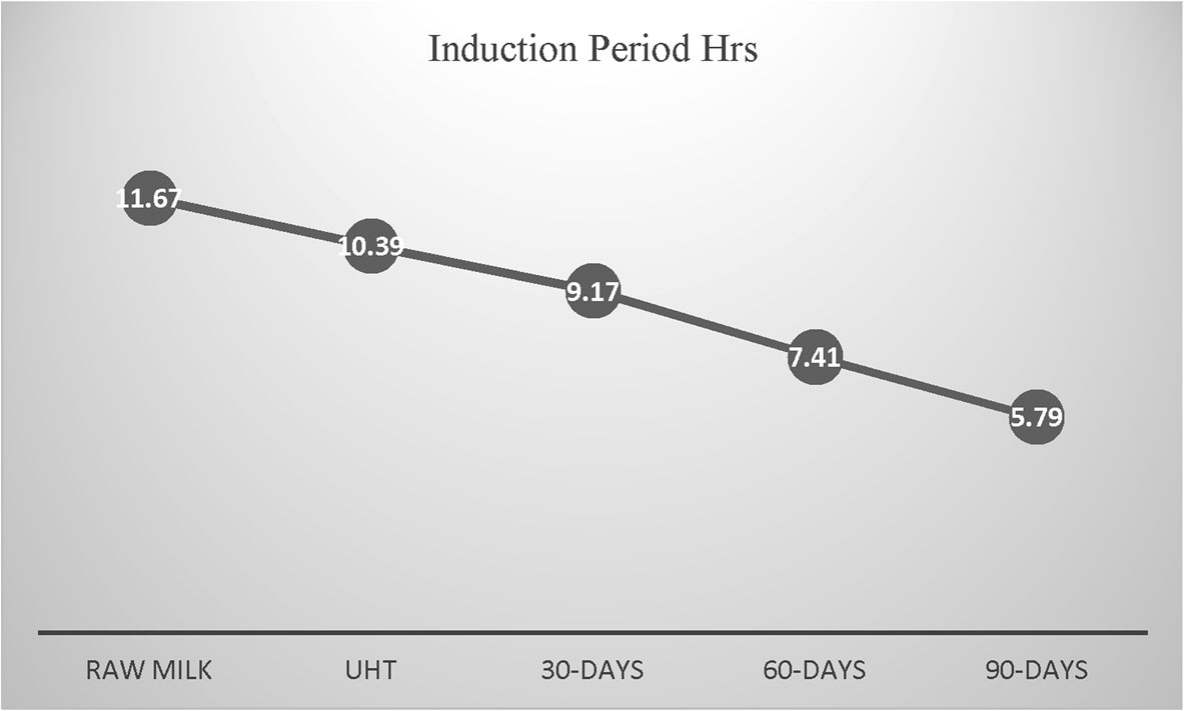
Induction Period of Raw, UHT Treated Milk in Storage

Induction period is used to estimate the oxidative stability index of fat and oils [6]. For the first time, this technique was used for the shelf life assessment of UHT milk. Research and development sections of dairy manufacturers have to wait for long time to know the oxidative stability of milk fat. The suitability of accelerated oxidation conditions to assess the oxidative stability of milk fat has already been established. For the shelf life assessment UHT milk, this technique is not previously for the measurement of oxidative stability of lipid phase of UHT milk. Induction period of raw, UHT and stored milk was strongly correlated with peroxide value and fatty acid profile. Induction period of milk samples was in the order of raw milk > UHT treated > 30 days old > 60 days old > 90 days old (Fig. 5). Measurement frequencies showing lower magnitude of oxidation products yielded high induction period. Induction period is influenced by the presence of antioxidants in food systems [52]. Milk also contains natural antioxidants which have been divided in two classes. First class is consisted of fat soluble antioxidants such as, vitamin E, A, carotene. Second class is comprised of water soluble substances which has antioxidant prospects. These include casein, whey proteins, vitamin C, amino acids, glutathione peroxidase and [31]. The lower values of induction period also indicated the weakening of antioxidant systems of milk during the storage. Strong correlations between the fatty acid profile and induction period was also recorded. Determination interval showing excessive breakdown of fatty acids had lower induction periods. These results offer promising opportunities for using induction period a useful method for the estimation of shelf life of UHT milk.

### Lipase activity

This is the first study in which lipase activity and triglyceride profile of milk has been investigated for a period of 3 months. Quality of raw milk in developing countries is very poor, total plate count in the months of summer may be as high 50million/ml. Lipases are fat hydrolyzing enzymes, delayed chilling of raw milk, excessive agitation and rough handling of milk may lead to increased lipolytic activity. Lipases of milk origin are usually eliminated by the UHT treatment but the lipases of bacterial origin are heat resistant, they survive the traditional UHT process and cause serious problems in lipid fraction of UHT milk during the storage. Mean value of lipase activity in raw milk was 0.73 ± 0.06 μmoles/ml. UHT treatment significantly decreased the lipase activity. The lipase activity of UHT treated milk was 0.44 ± 0.030.95 ± 0.07 and 1.14 ± 0.09 μmoles/ml. Lipase activity of UHT milk samples stored for 30, 60 and 90 days was 0.95 ± 0.07 and 1.14 ± 0.09 μmoles/ml. Lipase activity was strongly correlated with the triglyceride profile and free fatty acids. Testing intervals showing higher lipase activity also showed the higher concentration of free fatty acids. During the storage, triglycerides of UHT milk decreased and all the testing intervals showed a decreasing trend. During ambient storage, the increased lipolytic activity may be connected to the increased lipase activity [53]. Lipases produced by the *pseudomonas* spp. and *Bacillus* spp. are thermostable and survive the orthodox UHT treatment [54].

### Sensory evaluation

Results of sensory evaluation have been shown in Table 5. Color and smell score of UHT treated milk was significantly lower than raw milk (*p* < 0.05). Color, flavor and smell score decreased through the storage of UHT milk for 90 days. After 90 days of storage duration, color, flavor and smell score of UHT treated milk was 6.5, 6.3 and 6.2. The lower score of UHT milk during the storage was due to the changes taking place in fatty acid, triglyceride profile, organic acids, free fatty acids and oxidation products significantly affected the sensory characteristics of UHT milk. Lipase activity, free fatty acid and peroxide value of 90 days stored UHT milk was considerably higher, which was the reason for lower score of 90 days old samples of UHT milk.

**Table 5:** Effect of UHT Treatment and Storage on Sensory Characteristics of Milk

Stage	Color	Flavor	Smell
Raw Milk	8.1 ± 0.18^a^	ND	8.0 ± 0.15^a^
UHT-Treated	7.5 ± 0.09^b^	7.4 ± 0.12^a^	7.8 ± 0.10^a^
30 Days Old	7.3 ± 0.13^b^	7.1 ± 0.14^b^	7.2 ± 0.16^b^
60 Days Old	7.0 ± 0.15^b^	6.6 ± 0.22^b^	6.8 ± 0.16^b^
90 Days Old	6.9 ± 0.21^c^	6.5 ± 0.08^c^	6.7 ± 0.19^c^

In a column, if means are expressed by a different letter, that are statistically significant (*p* < 0.05)

^ND^Not Determined

## Conclusions

The results of this investigation disclosed that fatty acid and triglyceride profile of milk raw and 60 days old UHT milk samples were significantly different from each other. During the storage, lipase activity went on increasing which led to the transition and triglyceride profile and formation of free fatty acids. Peroxide value was strongly correlated with induction period and samples having lower peroxide value showed higher induction period and vice versa. Overall, induction period may be used for the assessment of oxidative stability of UHT milk.
